# Floor Covering for Hospitals

**Published:** 1904-01-30

**Authors:** 


FLOOR COVERING FOR HOSPITALS.

THE HON. SYDNEY HOLLAND writes: Some months ago I wrote, in answer to an inquiry in THE HOSPITAL, that, in my opinion, founded on some experience, linoleum was the best flooring for a hospital. I added "Cork linoleum must be avoided." I find that this sentence has done injustice to Messrs. Catesby, of Tottenham Court Road, who make a linoleum like the Staines and other linoleums, and call it "Cork Linoleum." What I meant to warn people against using for hospital floors was "Cork Carpet," as, though very suitable for private houses, it is more absorbent than linoleum (which is unabsorbent), and therefore not fit for hospital floors. I should be obliged if you would publish this in fairness to Messrs. Catesby, some of whose linoleum is in use at the London Hospital.

